# Optimising the therapeutic response of statins using real‐world evidence and machine learning: Personalised precision dosing recommends lower statin doses for some patients

**DOI:** 10.1111/dom.16029

**Published:** 2024-11-08

**Authors:** Andrew Krentz, Lisa Fournier, Thomas Castiglione, Vasa Curcin, Camil Hamdane, Tianyi Liu, André Jaun

**Affiliations:** ^1^ Metadvice Lausanne Switzerland; ^2^ School of Life Course & Population Sciences King's College London London UK; ^3^ Ecole Polytechnique Fédérale de Lausanne Lausanne Switzerland

**Keywords:** lipid‐lowering therapy, pharmaco‐epidemiology, primary care, real‐world evidence

## 1 BACKGROUND

Evidence‐based clinical guidelines for lipid modification are based on interventional clinical trials conducted in selected cohorts of patients according to predefined and restricted eligibility criteria.^1^ It follows that guidelines that are applicable to most patients, that is, those with similar characteristics to trial participants, might not be ideal for all patients. In the quest to achieve patient‐oriented outcomes that are as good as possible, prescribers may elect to intentionally deviate from published guidance.^2^ A recent study using machine learning applied to real‐world retrospective data from a Northern California health system reported that moderate‐ or low‐intensity statin therapy achieved better surrogate outcomes for a substantial minority of patients compared with high‐intensity statins.^3^ We tested the hypothesis that patients can be identified from UK primary care electronic health records for whom personalised cholesterol‐lowering therapy might be more appropriate than guideline‐based prescribing. We also confirmed the portability of our machine learning technology in a separate clinical data set.

## 2 METHODS

First, we developed a neural network model to reproduce prevailing UK national guidelines for cholesterol lowering, that is, National Institute for Health and Care Excellence (NICE) CG67,^4^ with a prespecified level of accuracy. A simple feedforward neural network was optimised to minimise the binary cross‐entropy with an equal probability over all possible recommendations. Monte Carlo testing against the rule‐based outcomes finally achieved 99.7% accuracy in predicting the right therapy and 98.1% accuracy to both predict the right therapy and none of the alternatives, leaving a neural network that evaluates adherence to guidelines with high accuracy. We then applied a transfer learning procedure to refine the clinical knowledge with real‐world evidence outcomes recorded in the UK Clinical Practice Research Datalink (CPRD),^5^ associating every therapeutic intervention with a non‐high‐density lipoprotein (non‐HDL) cholesterol reduction target. Data were split into 65% for training, 35% for testing/validation. Using artificial intelligence (AI) that combined knowledge from guidelines and real‐world evidence, we identified minority ‘digital twin’ cohorts likely to benefit from individualisation of cholesterol‐lowering therapy. A game theory concept known as Shapley values^6^ and the kernel SHapley Additive exPlanations approximation^7^ provided a measure of similarity to quantify the potential benefit of departing from the NICE guidelines by rejecting the no‐benefit hypothesis with a proportion test at *p* = 0.05 or 95% confidence level. Having established the neural network capabilities using the CPRD data set, an additional validation test studied the portability of the neural network into a clinical setting from South London, comprising 949 therapy decisions.

## 3 RESULTS

The CPRD sample with complete records who were receiving statin therapy comprised 9675 adult patients (mean ± SD age 74 ± 11 years; M 54% vs. F 46%; 86% White or not stated ethnicity with 4% South Indian, 3.3% Black, 2.9% Asian and 1.6% classified as other ethnicities; primary prevention vs. secondary prevention, 65% vs. 35%). Major comorbidities, that is, hypertension (71%) and type 2 diabetes (21%), were similar in prevalence between the primary and secondary prevention cohorts (data not shown).

**FIGURE 1:**
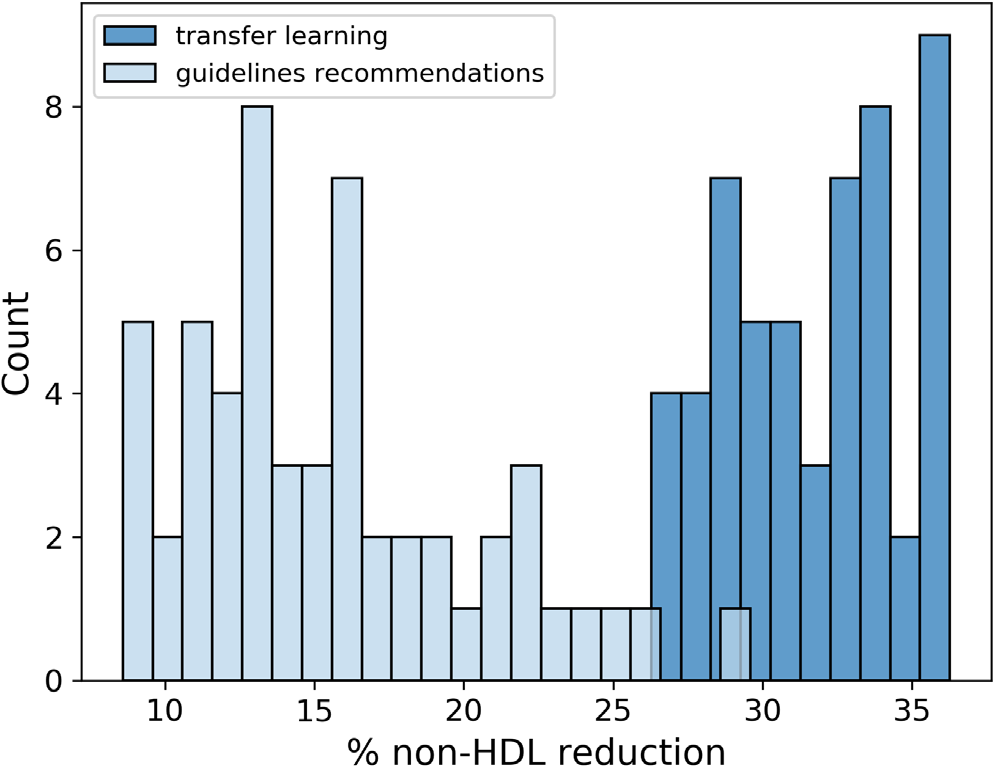
Histogram showing the average non‐high‐density lipoprotein (non‐HDL) cholesterol reduction obtained in the test set for digital twin cohorts optimised for a 25% target, in cases where the prescriber's decision was to administer guidelines (light blue) or the individualised medicine alternative (dark blue).

A broad distribution of responses in the primary outcome of interest, that is, non‐HDL cholesterol reduction, was observed, including a majority below the 40% guidance target and even paradoxical increases in some patients (data not shown). Using the median non‐HDL reduction observed in CPRD of 25% as an optimisation target, in the CPRD cohort the neural network generated two superposed histograms measuring the average non‐HDL cholesterol reduction outcomes for digital twin cohorts from the test data set where the clinician either followed guidelines or happened to choose the same therapy as the neural network recommended (Figure 1). This demonstrates that the clinical outcomes are not evenly distributed in Shapley value space and that the methodology has clear forecasting power.

Learning from real‐world outcomes, the model found that for up to 20% of patients, smaller statin doses achieved better lowering of non‐HDL cholesterol than doses recommended by the national guidelines. In the portability validation in six South London primary care clinics, all individualised recommendations suggesting a reduction in statin dosage had *p*‐values <0.05.

## 4 CONCLUSIONS

Our proof‐of‐concept study, performed in patient samples that are representative of the UK primary care population, supports the contention that machine learning can identify subgroups for whom smaller statin doses deviating from clinical guidelines may be associated with greater degrees of cholesterol lowering. These results, which require further prospective validation, provide clinicians with an actionable basis for a more individualised precision approach to cholesterol‐lowering pharmacotherapy. Our findings, based on independently developed and tested hypotheses, echo those of Sarraju et al.^3^

If sustained over time, failure to reduce non‐HDL cholesterol levels to evidence‐based goals may lead to avoidable cardiovascular events. Although an explanation for better cholesterol lowering using smaller statin doses cannot be determined from our analysis, a plausible mechanism is that adherence to therapy is better reflecting lower rates of statin‐associated adverse effects.^8^ This is testable in prospective cohort studies. Of potential relevance to this hypothesis, paradoxical increases in non‐HDL cholesterol were observed in a proportion of patients consistent with suboptimal adherence to medication.^9^ Of note, heterogeneity of therapeutic response is to be expected in our analysis. Cohorts identified as more optimally treated with lower intensity statin regimens may contain individuals who respond differently to a specified statin dose.

The strengths of our study include: first, the debiasing and portability that is achieved when combining two potentially biased sources of information (guidelines, real‐world data). Second, direct calculation of statistical support to inform clinical decisions using retrospective data permits an individualised clinical trial to be performed independent of a black‐box technology. Potential limitations of the study include well‐recognised deficiencies in the completeness of electronic health record coding and the restricted generalisability of the findings to populations outside the UK.

## CONFLICT OF INTEREST STATEMENT

Andrew Krentz and André Jaun are shareholders in Metadvice.

### PEER REVIEW

The peer review history for this article is available at https://www.webofscience.com/api/gateway/wos/peer‐review/10.1111/dom.16029.

## Data Availability

CPRD data are commercially available. Specific extracts reflect the contract between King's College London and CPRD.
